# Anti-Inflammatory Dysidazirine Carboxylic Acid from the Marine Cyanobacterium *Caldora* sp. Collected from the Reefs of Fort Lauderdale, Florida †

**DOI:** 10.3390/molecules27051717

**Published:** 2022-03-06

**Authors:** Sarath P. Gunasekera, Sofia Kokkaliari, Ranjala Ratnayake, Thomas Sauvage, Larissa A. H. dos Santos, Hendrik Luesch, Valerie J. Paul

**Affiliations:** 1 Smithsonian Marine Station, 701 Seaway Drive, Ft. Pierce, FL 34949, USA; gunasekeras@si.edu (S.P.G.); tomsauv@gmail.com (T.S.); lari.akiko@gmail.com (L.A.H.d.S.); 2 Department of Medicinal Chemistry and Center for Natural Products, Drug Discovery and Development (CNPD3), University of Florida, 1345 Center Drive, Gainesville, FL 32610, USA; skokkaliari@cop.ufl.edu (S.K.); rratnayake@cop.ufl.edu (R.R.); luesch@cop.ufl.edu (H.L.); 3 Instituto de Biociências, Universidade Federal do Rio Grande do Sul, Porto Alegre 90650, RS, Brazil

**Keywords:** marine cyanobacteria, *Caldora*, Oscillatoriales, marine natural products, azirine natural product, cytotoxicity, anti-inflammatory activity, iNOs

## Abstract

Dysidazirine carboxylic acid (**1**) was isolated from the lipophilic extract of a collection of the benthic marine cyanobacterium *Caldora* sp. from reefs near Fort Lauderdale, Florida. The planar structure of this new compound was determined by spectroscopic methods and comparisons between HRMS and NMR data with its reported methyl ester. The absolute configuration of the single chiral center was determined by the conversion of **1** to the methyl ester and the comparison of its specific rotation data with the two known methyl ester isomers, **2** and **3**. Molecular sequencing with 16S rDNA indicated that this cyanobacterium differs from *Caldora* *penicillata* (Oscillatoriales) and represents a previously undocumented and novel *Caldora* species. Dysidazirine (**2**) showed weak cytotoxicity against HCT116 colorectal cancer cells (IC_50_ 9.1 µM), while dysidazirine carboxylic acid (**1**) was non-cytotoxic. Similar cell viability patterns were observed in RAW264.7 cells with dysidazirine only (**2**), displaying cytotoxicity at the highest concentration tested (50 µM). The non-cytotoxic dysidazirine carboxylic acid (**1**) demonstrated anti-inflammatory activity in RAW264.7 cells stimulated with LPS. After 24 h, **1** inhibited the production of NO by almost 50% at 50 µM, without inducing cytotoxicity. Compound **1** rapidly decreased gene expression of the pro-inflammatory gene *iNOS* after 3 h post-LPS treatment and in a dose-dependent manner (IC_50_ ~1 µM); the downregulation of *iNOS* persisted at least until 12 h.

## 1. Introduction

**Figure 1:**
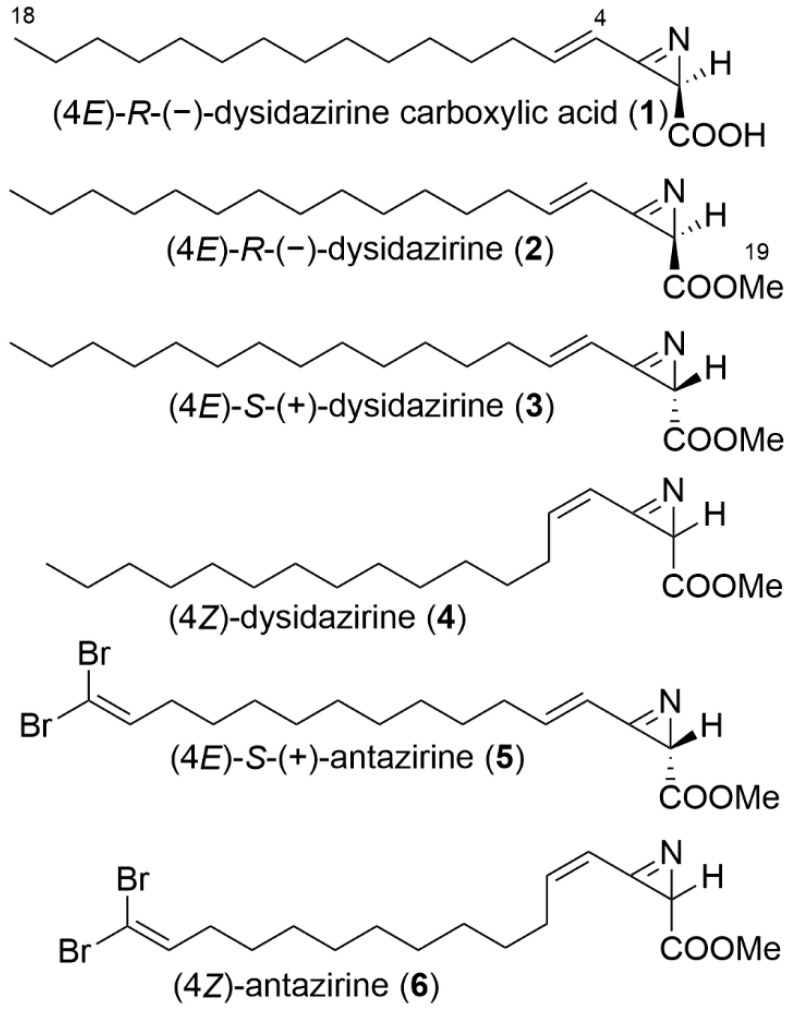
Chemical structures of azirine natural products.

Azirine natural products are rare in nature. Azirinomycin is the first example of a natural product containing an unstable strained azirine ring. It was discovered in 1971 from a cultured broth of a strain of *Streptomyces aureus* from a soil sample [1]. Azirinomycin has a simple structure of 3-methyl-2*H*-azirine-2-carboxylic acid. Azirinomycin and its semi-synthetic methyl ester exhibit broad spectrum in vitro antibiotic activity against both Gram-positive and Gram-negative bacteria [2]. Molinski and Ireland reported the isolation of cytotoxic dysidazirine (**2**), an azacyclopropene methyl ester, from the marine sponge *Dysidea fragilis* collected in Fiji, and this was the first example of a strained 2H-azirine from a marine source [3]. In 1995, Faulkner’s group reported the isolation of four azacyclopropane methyl esters (**3**–**6**) related to dysidazirine (**2**) from the same marine sponge *Dysidea fragilis* collected in Pohnpei, the Federated States of Micronesia [4] (Figure 1). Compound (**2**), (4*E*)-*R*-dysidazirine, was previously shown to be active against *Pseudomonas aeruginosa, Candida albicans*, and *Saccharomyces cerevisiae* [3,5,6]. It was also shown to possess activity against liquid tumor cells, with an IC_50_ of 0.88 µM against the L1210 (mouse lymphocytic leukemia) cell line. Activity against cancer cell lines derived from solid tumors has not been reported for this compound; however, halogenated analogues have shown activity against the human colon cancer cell line HCT116 [3,5,6]. Dysidazirine (**2**) has received attention from chemists and inspired the synthesis of new antibacterial and antifungal derivatives [7,8]. Structure activity studies of synthetic dysidazirine analogues indicated that antifungal activity was dependent on the C4-C5 unsaturation but was not strongly dependent on the configuration at C2 or minor changes in chain lengths. Branching of the terminal end abolished antifungal activity [7].

Here, we report the first isolation of 2H-azirine free carboxylic acid (**1**), a possible biosynthetic intermediate of dysidazirine (**2**), from a cyanobacterium *Caldora* sp. collected from a reef near Fort Lauderdale, Florida. The existence of biosynthetic pathways to produce rare azirines in the terrestrial bacterium *Streptomyces aureus*, the marine sponge *Dysidea fragilis*, and marine cyanobacterium *Caldora* sp. suggest a possible microbial origin for the compounds in the sponge.

## 2. Results and Discussion

### 2.1. Isolation and Structure Elucidation

The sample of the marine cyanobacterium *Caldora* sp. was collected in shallow water (7–10 m depth) while scuba diving near Fort Lauderdale, Florida, in July 2017, and was repeatedly present at this location near a coral reef monitoring site during the summer months in 2017–2018, resulting in several recollections. The samples were transported live in seawater in a cooler to the Smithsonian Marine Station at Fort Pierce, Florida, where they were drained, frozen, and freeze-dried. Freeze-dried material was extracted with a mixture of EtOAc-MeOH (1:1) to afford a lipophilic extract, which was subsequently partitioned between EtOAc and H_2_O. The EtOAc-soluble portion was fractionated by SiO_2_ column chromatography, followed by reversed-phase C18 column chromatography, and then further purified by reversed-phase HPLC to give the new compound, dysidazirine carboxylic acid (**1**) (Figure 1).

Dysidazirine carboxylic acid (**1**) was obtained as a colorless solid. The molecular formula C_18_H_31_NO_2_ was determined from HRESIMS data. The appearance of a strong negative ion at *m/z* 292.2276 for [M-H]^−^ and the presence of a single carbonyl signal at 176.2 ppm in the ^13^C spectrum suggested the compound to be an acid. To confirm the presence of a free carboxylic acid, **1** was reacted with trimethylsilyldiazomethane, yielding the methyl ester (**2**). HRESIMS data gave the expected molecular formula of C_19_H_33_NO_2_ for the methyl ester (**2**).

Following the interpretation of DQF ^1^H-^1^H COSY, edited HSQC and HMBC experiments (Table 1) (Supplementary Materials S2–S6), the ^1^H and ^13^C NMR signals of **1** were assignable to one terminal methyl group (C-18, *δ*_H_ 0.89, *δ*_C_ 14.4), twelve consecutive methylene groups, three conjugated olefinic carbons (C-3 *δ*_C_ 157.1; C-4 *δ*_H_ 6.61, *δ*_C_ 114.1; C-5 *δ*_H_ 6.75, *δ*_C_ 158.4), one methine carbon as a singlet (C-2, *δ*_H_ 2.49, *δ*_C_ 29.4), and one carbonyl carbon C-1 (*δ*_C_ 176.2). The COSY spectrum connected the alkyl chain to the double bond via single allylic methylene group (C-6, *δ*_H_ 2.38, *δ*_C_ 34.1). The two coupled proton signals C-4 at 6.61 ppm (d, *J* = 15.1 Hz) and C-5 at 6.75 ppm (dd, *J* = 15.1, 6.9 Hz) were assignable to a *trans*-disubstituted olefin. HMBC correlations indicated H-2 (*δ*_H_ 2.49) to C-1 carbonyl group (*δ*_C_ 176.2), C-3 (*δ*_C_ 157.1) and C-4 (*δ*_C_ 114.1), and this information connected the acid terminal end to the alkyl chain via the double bond. Dereplication using the molecular formula of the methyl ester **2** in the marine natural product database, together with the ^1^H and ^13^C NMR spectral data analysis, identified the prepared methyl ester **2** of the isolate as one of the three previously reported dysidazirines (**2**–**4**) [3,4]. This information, together with the ^1^H-^1^H COSY, HMBC, and HSQC NMR data analysis, particularly the coupling constant of *J* = 15.1 Hz for 4-H and 5-H of the free acid (**1**) and the methyl ester (**2**), eliminated the (*Z*) geometric isomer (**4**). This limited the two enantiomers to (4*E*)-*R*-dysidazirine (**2**) and (4*E*)-*S*-dysidazirine (**3**). Comparison of the observed negative optical rotation data of the prepared methyl ester [α]^25^D −166.7 (*c* 0.06, CDCl_3_) with the reported optical data of the two enantiomers identified the prepared methyl ester as (4*E*)-*R*-dysidazirine (**2**); literature value [α]D −165 (*c* 0.5, MeOH), first reported as dysidazirine [3]. In contrast, the reported optical rotation for (4*E*)-*S*-dysidazirine (**3**) was positive ([α]D +47.2 (*c* 1.08, CDCl_3_)) [4]. With only one stereocenter, this confirmed the structure of the isolated precursor acid as (4*E*)-*R*-dysidazirine carboxylic acid (**1**).

### 2.2. Identification of the Cyanobacterium

**Figure 2:**
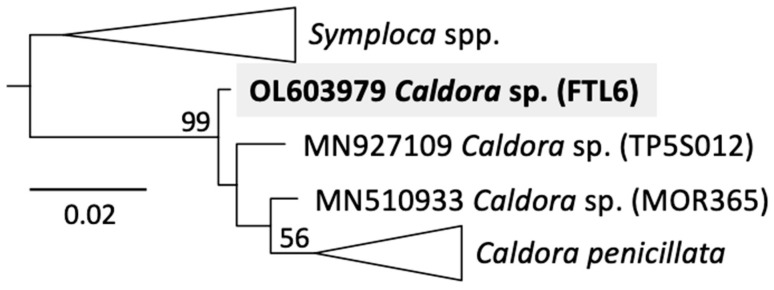
16S rDNA maximum-likelihood tree showing the early branching phylogenetic position of the sample FTL6 within *Caldora* spp. The numerous sequences found in the *C. penicillata* clade, as delimited by Engene et al. [10], are summarized as a triangle for figure clarity (see Supplementary Materials Figure S7 for additional details).

DNA extraction, sequencing, and phylogenetic analyses were conducted, as previously described [9]. Phylogenetic reconstruction based on 16S rDNA identified our *Caldora* sample, FTL6 collected in Fort Lauderdale on 16 October 2017, as an undocumented and novel sequence for this genus (Figure 2). Our tree showed three sequences that branch prior to the clade delimited as *Caldora penicillata* by Engene et al. [10], which receives overall low bootstrap support (56%), possibly due to sequences swapping position within this clade during tree reconstruction (see Supplementary Materials Figure S7 for the poor topology within this clade caused by the overall variability of 16S rDNA sequences). However, the *Caldora* genus was strongly supported, with FTL6 consistently branching first (99% node bootstrap support) (Figure 2).

**Figure 3:**
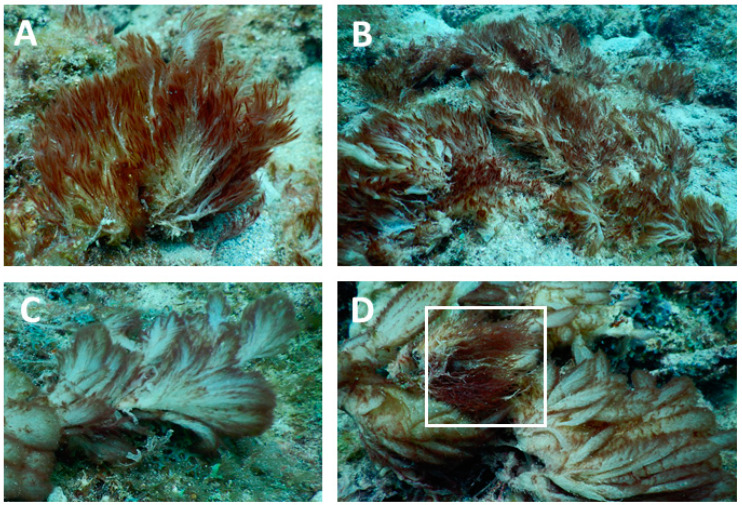
Underwater photographs of *Caldora* sp. in comparison to *Caldora penicillata* on the reef near Fort Lauderdale, FL. (**A**,**B**) Macroscopic photographs of tufts of *Caldora* sp. Note the distinct morphology of *Caldora* sp., as the filaments are intertwined giving it a “stringy” appearance. (**C**) *Caldora penicillata* [10]. (**D**) The two species can also grow together, and the white box shows *Caldora* sp. as it overgrows *Caldora penicillata*.

Considering its unique and distinctive morphology (Figure 3), the novel chemistry of *Caldora* sp. FTL6, and its consistent phylogenetic branching prior to *C. penicillata*, we consider this specimen as a novel species within the genus *Caldora*. For further confirmation, future endeavors should reassess genus diversity with more variable markers than 16S rDNA (e.g., such as the protein encoding gene *tufA*) to improve topological features of the tree (Figure 2).

### 2.3. Biological Activity

**Figure 4:**
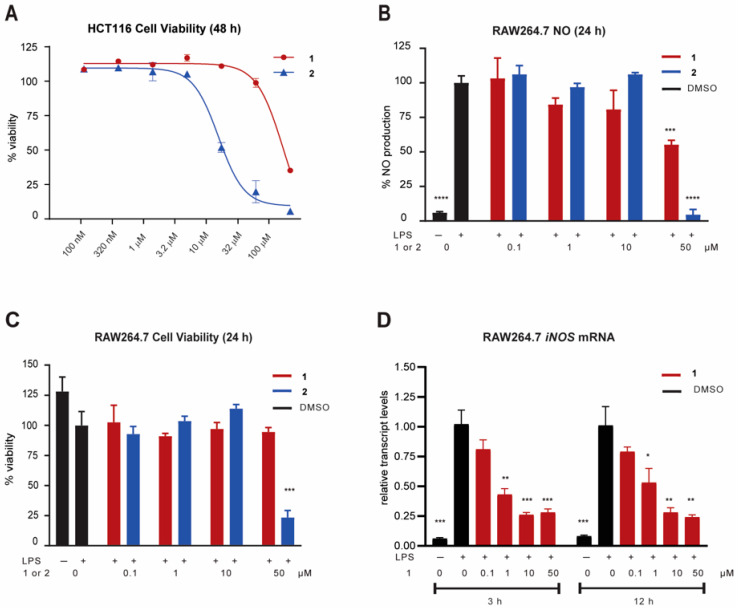
Bioactivity of (4*E*)-*R*-dysidazirine carboxylic acid (**1**) and methyl ester **2.** (**A**) Antiproliferative activity against human colon cancer cells (HCT116) measured by MTT assay at 48 h. Gatorbulin-1 was used as a positive control, tested at the same time (IC_50_ 0.80 μM) [11]. (**B**) Anti-inflammatory activity of pretreatment for 1 h with **1**, **2** (50, 10, 1 and 0.1 μM), or vehicle control (0.5% DMSO) by measuring the production of nitric oxide (NO) in murine macrophages (RAW264.7) 24 h after LPS stimulation. (**C**) Cell viability of RAW264.7 cells using the MTT assay at 24 h under the same conditions used in the NO assay. (*D*) *iNOS* target gene expression in RAW264.7 after pretreatment for 1 h with **1** or vehicle control (0.5% DMSO prior to LPS addition for 3 and 12 h). RNA was isolated, reverse-transcribed, and subjected to qPCR, using *β*-actin as the endogenous control. The values were normalized to vehicle control treated with LPS for each time point. Non-stimulated cells (no LPS) were tested simultaneously (**B**–**D**). Error bars indicate the mean ± SD of three replicates for graphs B, C, and D. Statistical analysis was performed using multiple comparison t-tests (* *p* < 0.05, ** *p* < 0.01, *** *p* < 0.001, **** *p* < 0.0001, compared to LPS treatment alone).

Carboxylic acid **1** and its methyl ester **2** were tested against human colon cancer HCT116 cells to probe for antiproliferative activity. The cell viability assay indicated modest antiproliferative activity of **2** (IC_50_ 9.1 µM) and 8.8-fold reduced activity of **1** (79.7 µM) (Figure 4A). Compounds **1** and **2** were then tested for potential anti-inflammatory activity by measuring nitric oxide (NO) production in RAW264.7 cells stimulated by LPS. Cells were pretreated with compounds **1** and **2** separately for 1 h and then challenged with LPS. After 24 h, acid **1** inhibited the production of NO by almost 50% at 50 µM, without inducing cytotoxicity at that concentration, while methyl ester **2** was cytotoxic at that concentration, leading to apparent NO inhibition (Figure 4B,C). The increased cytotoxicity of **2** versus **1** was consistent with our data in HCT116 cells. Therefore, we focused on compound **1** for further mechanistic studies related to NO inhibition and anti-inflammatory activity at non-cytotoxic concentrations. To determine if NO inhibition might be a result of the transcriptional regulation of inducible nitric oxide synthase (*iNOS*), the NF-κB target gene encoding the synthesis of NO, we measured the transcript levels of this pro-inflammatory gene. Compound **1** rapidly decreased gene expression after 3 h post-LPS treatment and in a dose-dependent manner; the downregulation of *iNOS* persisted at least until 12 h (Figure 4D). Compound **1** at 3 h and 12 h showed a similar response, with the target gene showing a 50% reduction near 1 µM and roughly 75% reduction at 10 and 50 µM. Therefore, early transcriptional changes were even more sensitive to compound **1** than functionally-linked NO levels after 24 h.

## 3. Materials and Methods

### 3.1. General Experimental Procedures

Optical rotations were recorded on a Rudolph Research Analytical Autopol III automatic polarimeter. UV spectrophotometric data were acquired on a Shimadzu PharmaSpec UV–visible spectrophotometer. NMR data were collected on a JEOL ECA-600 spectrometer operating at 600.17 MHz for ^1^H and 150.9 MHz for ^13^C. The edited-HSQC experiment was optimized for *J*_CH_ = 140 Hz and the HMBC spectrum was optimized for ^2/3^*J*_CH_ = 8 Hz. ^1^H NMR chemical shifts (referenced to residual CHCl_3_ observed at *δ* 7.25 and residual CH_3_OH observed at *δ* 3.30) were assigned using a combination of data from 2D DQF COSY and multiplicity-edited HSQC experiments. Similarly, ^13^C NMR chemical shifts (referenced to residual CHCl_3_ observed at *δ* 77.0 and residual CH_3_OH observed at *δ* 49.0) were assigned based on the multiplicity-edited HSQC experiments. The HRMS data were obtained using an Agilent 6210 LC-TOF mass spectrometer equipped with an APCI/ESI multimode ion source detector at the Mass Spectrometer Facility at the University of California, Riverside, California. Silica gel 60 (EMD Chemicals, Inc., Port Wentworth, GA, USA, 230–400 mesh) was used for column chromatography. All solvents used were of HPLC grade (Fisher Scientific, Waltham, MA, USA).

### 3.2. Collection, Extraction and Isolation

The first sample of *Caldora* sp. for this study was collected in July 2017 from Fort Lauderdale, Florida. The sample was transported in seawater in a cooler to the Smithsonian Marine Station at Ft. Pierce, Florida, where it was drained, frozen in a plastic bag, and then freeze-dried. The freeze-dried material (48.5 g) was repeatedly extracted in a glass beaker with EtOAc-MeOH (1:1) to give 4.93 g of the lipophilic extract. This extract was partitioned between EtOAc and H_2_O. The EtOAc-soluble fraction (1.04 g) was chromatographed on a column of SiO_2_ (25 g) using a step gradient system of hexanes-EtOAc, EtOAc, EtOAc-MeOH, and EtOAc-MeOH to give four sub-fractions. The second sub-fraction that was eluted with EtOAc (0.080 g) was further chromatographed on a column of C_18_ (4 g) using a step gradient system of MeOH-H_2_O, MeOH-H_2_O, and MeOH to give 3 sub-fractions. The second sub-fraction (0.021 g) was further purified by reversed-phase HPLC (semi-prep 250 × 10 mm, 5 μm, RP-18, flow 3.0 mL/min) using MeOH-12% H_2_O to give dysidazirine carboxylic acid (**1**) (3.0 mg, *t*_R_ = 19.8 min, yield, 0.006% dry wt).

*Dysidazirine carboxylic acid* (**1**): colorless, solid; [α]^25^D 145.2 (*c* 0.12, CDCl_3_); UV(MeOH) λ_max_ (log *ε*) 222 (4.11) nm; ^1^H and ^13^C NMR data (see Table 1) assignments were made by the interpretation of 2D DQF COSY, edited-HSQC, and HMBC data (Supplementary Materials S2–S6); HRESI/TOFMS *m*/*z* 292.2276 [M-H]^−^ (calcd for C_18_H_30_NO_2_, 292.2278).

For the methylation of dysidazirine carboxylic acid (**1**), compound **1** (1.0 mg) was dissolved in 1.0 mL of MeOH and cooled to 4 °C. The cooled solution was treated with 50 μL of 2.0 M trimethylsilyl diazomethane in diethyl ether. Evaporation of MeOH and excess trimethylsilyl diazomethane gave an oily product. This product was purified by reversed-phase HPLC (semi-prep 250 × 10 mm, 5 μm, RP-18, flow 3.0 mL/min) using MeOH-5% H_2_O to give methyl ester (4*E*)-*R*-dysidazirine **2** (0.9 mg, *t*_R_ = 14.4 min).

(4*E*)-*R*-dysidazirine (**2**): colorless, solid; [α]^25^D -166.7 (*c* 0.06, CDCl_3_), [lit. [α]D -165 (*c* 0.5, MeOH)[4]; UV (MeOH) λ_max_ (log *ε*) 222 (4.11) nm; ^1^H and ^13^C NMR data, see Table 1; HRESI/TOFMS *m*/*z* 308.2613 [M-H]^−^ (calcd for C_19_H_34_NO_2_, 308.2590).

### 3.3. Molecular Identification

One of multiple recollections over a 2-year period (2017–2018), the sample used for sequencing of the 16S rRNA gene (FTL6) was collected in Fort Lauderdale on 16 October 2017 in the same coral monitoring site as the original collection (see above). A voucher specimen is maintained at the Smithsonian Marine Station, Fort Pierce, FL. DNA extraction, sequencing, and phylogenetics were conducted as previously described [9].

### 3.4. Cancer Cell Viability Assay (HCT116)

Human colon cancer cells HCT116 were used as a representative model system to probe for antiproliferative activity. HCT116 cells were cultured in Dulbecco’s modified Eagle’s medium (DMEM), supplemented with 10% fetal bovine serum at 37 °C humidified air and 5% CO_2_. Cells were seeded (8000 cells/well) in 96-well plates, allowed to attach overnight and treated with (4*E*)-*R*-dysidazirine carboxylic acid (**1**), methyl ester (**2**), and the solvent control (0.5% DMSO). Cell viability was measured after 48 h, following treatment with MTT dye using the manufacturer’s protocol (Promega, Madison, WI, USA). IC_50_ values were calculated from variable slope fitting for a dose response curve using GraphPad Prism software. Gatorbulin-1 was tested at the same time (IC_50_ 0.80 μM) [11] and served as a positive control.

### 3.5. NO Assay and Cell Viability Assay (RAW264.7 Cells)

RAW264.7 cells were cultured in Dulbecco’s modified Eagle’s medium (DMEM), supplemented with 10% fetal bovine serum at 37 °C humidified air and 5% CO_2_. Cells were seeded (2 × 10^4^/well) in 96-well plates and allowed to attach for 24 h before being treated with the compounds (**1** and **2**) or the solvent control (0.5% DMSO) for 1 h, followed by the addition of LPS at 1 μg/mL. Non-stimulated cells (no LPS) were tested simultaneously. The production of NO in the cell supernatant was measured after 24 h by measuring the nitrite concentration, which is an oxidative product of NO. A total of 50 μL of the supernatant was mixed with the Griess reagent using the manufacturer’s protocol (Promega) and the absorbance was measured at 540 nm. The nitrite concentration was derived from a calibration curve generated from a fresh nitrite standard solution. Cell viability was measured under the same seeding conditions and time points, using MTT dye following the manufacturer’s protocol (Promega).

### 3.6. iNOS mRNA Measurement in RAW264.7 Cells

RAW264.7 cells were seeded (2.5 × 10^5^/well) in 6-well plates and treated with different concentrations of **1** or vehicle control for 1 h prior to treatment with LPS at 1 μg/mL and then incubated for 3 and 12 h. Non-stimulated cells (no LPS) were tested simultaneously. Total RNA was extracted using the RNAeasy Mini Kit (QIAGEN, Germantown, MD, USA). RNA was reverse-transcribed to cDNA using SuperScript II Reverse Transcriptase (Invitrogen, Carlsbad, CA, USA) and oligo (dT) (Invitrogen), starting with 500 ng of total RNA. The cDNA samples were used as templates for TaqMan gene expression assays (Applied Biosystems, Waltham, MA, USA). The qPCR analysis was performed in triplicate at 25 µL total volume (12.5 μL of TaqMan 2× universal master mix, 1.25 μL of a 20× TaqMan gene expression assay probe, 1 μL of cDNA, and 10.25 μL of RNase-free sterile water) to detect the expression of *iNOS* (Mm00440502_m1, Applied Biosystems) and *β-actin* (internal standard, 4352663, Applied Biosystem). The qPCR method was as follows: 50 °C for 2 min, 95 °C for 10 min, and 40 cycles of 95 °C for 15 s and 60 °C for 1 min.

## Figures and Tables

**Table 1:** NMR spectroscopic data for (4*E*)-*R*-dysidazirine carboxylic acid (**1**) and (4*E*)-*R*-dysidazirine (**2**).

C No	*δ*_C_ Mult.	*δ*_H_ (*J* in Hz)	COSY	HMBC *^a^*	*δ*_C_ Mult.	*δ*_H_ (*J* in Hz)
	1 *^b^*				2 *^c^*^,*d*^	
**1**	176.2, C			2	172.2, C	
**2**	29.4, CH	2.49, s		4	28.3, CH	2.45, s
**3**	157.1, C			2, 4, 5	156.6, C	
**4**	114.1, CH	6.61, d (15.1)	5, 6	2, 5	112.9, CH	6.47, d (15.8)
**5**	158.4, CH	6.75, dt (15.1, 6.9)	4, 6	6, 7	155.8, CH	6.64, dt (15.8, 6.8)
**6**	34.1, CH_2_	2.38, dt (6.9, 7.4)	5, 7	4, 5, 7	33.2, CH_2_	2.28, dt (6.8, 7.5)
**7**	29.0, CH_2_	1.52, m	6, 8	5, 6	29.1, CH_2_	1.44, m
**8–15**	30.3–30.8, CH_2_	1.35–1.24, m			29.2–29.7, CH_2_	1.1–1.3, m
**16**	33.0, CH_2_	1.32, m		18	33.2, CH_2_	1.17, m
**17**	23.7, CH_2_	1.24, m	18	18	22.6, CH_2_	1.17, m
**18**	14.4, CH_3_	0.89, t (6.8)	17	16, 17	14.1, CH_3_	0.79, t (6.8)
**19**					52.2, CH_3_	3.34, s

*^a^* HMBC correlations, optimized for ^2/3^*J*_CH_ = 8 Hz, are from proton(s) stated to the indicated carbon. *^b^* In CD_3_OD. *^c^* Proton data in CD_3_OD with a few drops of CDCl_3_ to improve solubility. *^d^* Carbon data in CDCl_3_.

## Supplementary Materials

The following are available online: ^1^H, ^13^C, and 2D ^1^H-^1^H COSY, HSQC and HMBC NMR spectra in CD_3_OD for dysidazirine carboxylic acid (**1**), and ^1^H and ^13^C NMR spectra for (4*E*)-*R*-dysidazirine (**2**) in CDCl_3_-CD_3_OD and CDCl_3_, respectively. Fully developed 16S rDNA maximum likelihood tree showing *Caldora penicillata* sequences summarized as a triangle in Figure 2.

## Sample Availability

Samples of the compounds are available from the corresponding author.
