# Supplementary material for: Anti-Inflammatory Dysidazirine Carboxylic Acid from the Marine Cyanobacterium Caldora sp. Collected from the Reefs of Fort Lauderdale, Florida
Source: Molecules. 2022 Mar 6;27(5):1717. doi: 10.3390/molecules27051717 (PMC8911782; doi:10.3390/molecules27051717)
Supplement: Supplementary file 1 [file molecules-27-01717-s001.zip › molecules-1539671-supplementary.pdf]

Anti-inflammatory dysidazirine carboxylic acid from the marine cyanobacterium *Caldora sp.* collected from the reefs of Fort Lauderdale, Florida#

Sarath P. Gunasekera[1], Sofia Kokkaliari[2], Ranjala Ratnayake[2], Thomas Sauvage[1], Larissa A. H. dos Santos[1], Hendrik Luesch[2] and Valerie J. Paul[1*]

[1] Smithsonian Marine Station at Ft. Pierce, 701 Seaway Drive, Ft. Pierce, FL 34949, USA

[2] Department of Medicinal Chemistry and Center for Natural Products, Drug Discovery and Development (CNPD3), University of Florida, 1345 Center Drive, Gainesville, Florida 32610, USA

[3] Instituto de Biociendas, Universidade Federal do Rio Grande do Sul, Porto Alegre, RS, 90650, Brazil

* To whom correspondence should be addressed. E-mail: paul@si.edu

# Dedicated to Dr. William Fenical, University of California San Diego, for his pioneering work on bioactive natural products.

Contents

	Page #
^{1}H NMR (600 MHz, CD_3OD) spectrum of dysidazirine carboxylic acid (**1**)	S 2
^{13}C NMR (150 MHz, CD_3OD) spectrum of dysidazirine carboxylic acid (**1**)	S 3
COSY (600 MHz, CD_3OD) spectrum of dysidazirine carboxylic acid (**1**)	S 4
Edited HSQC (600 MHz, CD_3OD) spectrum of dysidazirine carboxylic acid (**1**)	S 5
HMBC (600 MHz, CD_3OD) spectrum of dysidazirine carboxylic acid (**1**)	S 6
JH NMR (600 MHz, $CDCI_3$ and CD_3OD) spectrum of dysidazirine (2)	S 7
^{13}C NMR (150 MHz, CDCla) spectrum of dysidazirine (2)	S 8
Fully developed 16S rDNA maximum likelihood tree for Caldora spp.	S 9

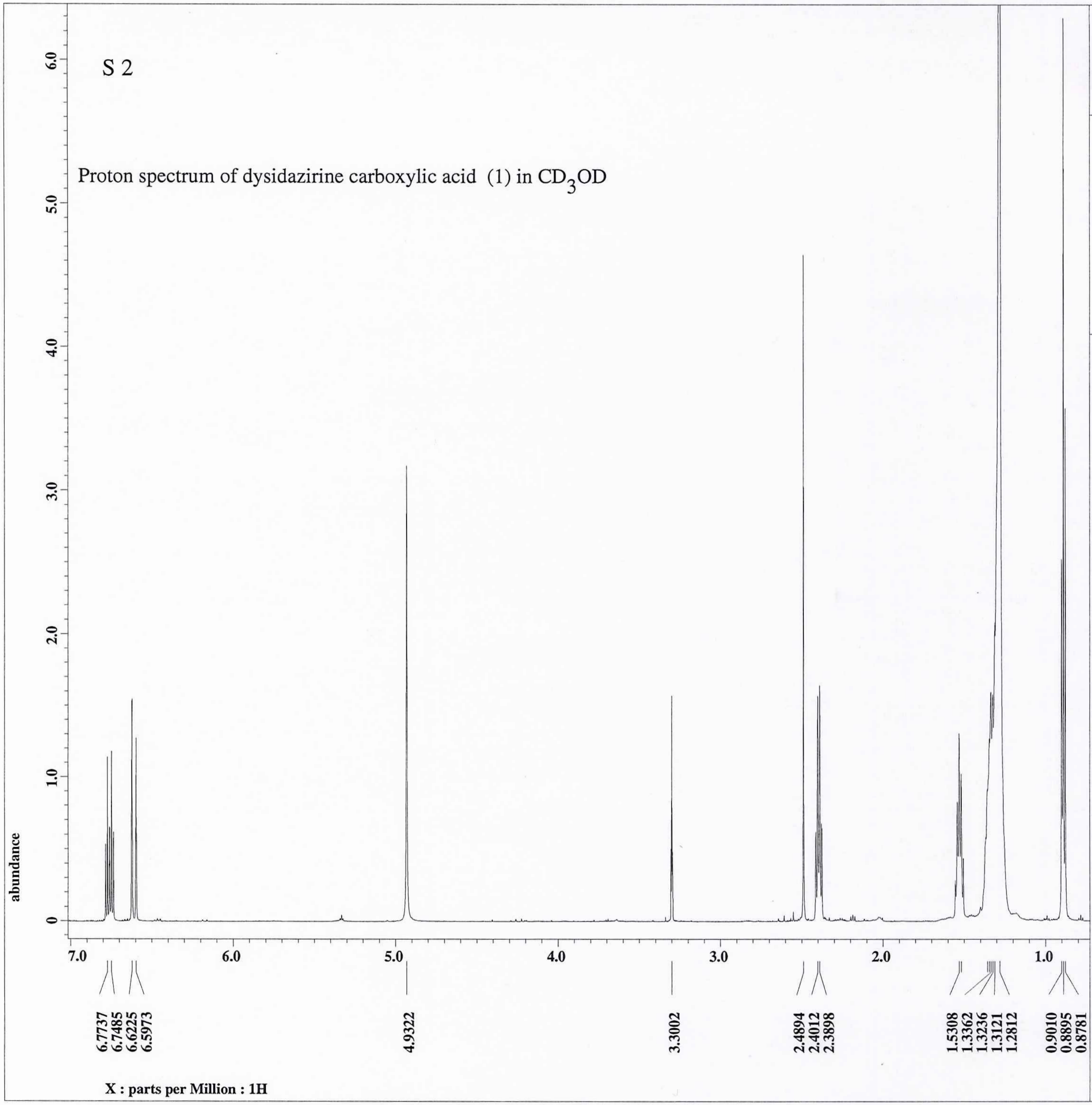

---- PROCESSING PARAMETERS ----
dc_balance : 0 : FALSE
sexp : 0.2[Hz] : 0.0[s]
trapezoid3 : 0[%] : 80[%] : 100[%]
zerofill : 1
fft : 1 : TRUE : TRUE
machinephase
ppm

Filename	= 6sg73-9-single_pulse-
Author	= Smithsonian
Experiment	= single_pulse.ex2
Sample_id	= 6sg73-9
Solvent	= METHANOL-D3
Creation_time	= 19-APR-2018 07:22:43
Revision_time	= 24-AUG-2021 14:13:22
Current_time	= 24-AUG-2021 14:14:30
Comment	= single_pulse
Data_format	= 1D COMPLEX
Dim_size	= 13107
Dim_title	= 1H
Dim_units	= [ppm]
Dimensions	= X
Site	= ECA 600
Spectrometer	= ECA600-AID
Field_strength	= 14.09636928[T] (600[M
X_acq_duration	= 1.4548992[s]
X_domain	= 1H
X_freq	= 600.1723046[MHz]
X_offset	= 5[ppm]
X_points	= 16384
X_prescans	= 1
X_resolution	= 0.68733284[Hz]
X_sweep	= 11.26126126[kHz]
Irr_domain	= 1H
Irr_freq	= 600.1723046[MHz]
Irr_offset	= 5[ppm]
Tri_domain	= 1H
Tri_freq	= 600.1723046[MHz]
Tri_offset	= 5[ppm]
Clipped	= FALSE
Mod_return	= 1
Scans	= 8
Total_scans	= 8
X_90_width	= 7.9[us]
X_acq_time	= 1.4548992[s]
X_angle	= 45[deg]
X_atn	= 3[dB]
X_pulse	= 3.95[us]
Irr_mode	= Off
Tri_mode	= Off
Dante_presat	= FALSE
Initial_wait	= 1[s]
Recvr_gain	= 34
Relaxation_delay	= 5[s]
Repetition_time	= 6.4548992[s]
Temp_get	= 22.4[dC]

S 3

Carbon spectrum of dysidazirine carboxylic acid (1) in CD_3OD

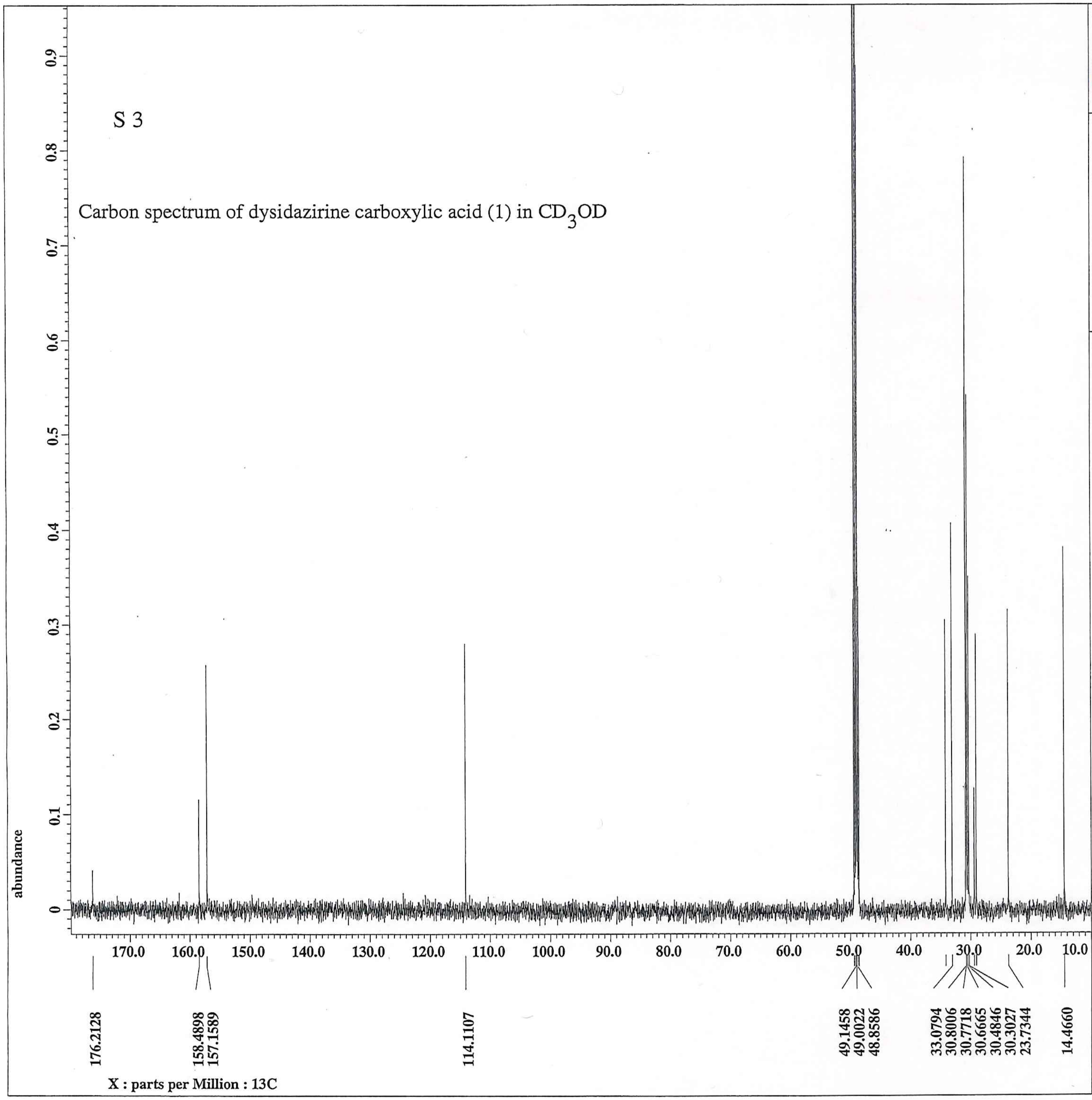

JEOL

```
---- PROCESSING PARAMETERS ----
dc_balance : 0 : FALSE
sexp : 2.0[Hz] : 0.0[s]
trapezoid3 : 0[%] : 80[%] : 100[%]
zerofill : 1
fft : 1 : TRUE : TRUE
machinephase
ppm

Filename            = 6sg73-9-single_pulse_
Author              = Smithsonian
Experiment          = single_pulse_dec
Sample_id           = 6sg73-9
Solvent             = METHANOL-D3
Creation_time       = 19-APR-2018 07:40:49
Revision_time       = 20-DEC-2021 10:08:27
Current_time        = 20-DEC-2021 10:10:07

Comment             = single pulse decouple
Data_format         = 1D COMPLEX
Dim_size            = 26214
Dim_title           = 13C
Dim_units           = [ppm]
Dimensions          = X
Site                = ECA 600
Spectrometer        = ECA600-AID

Field_strength      = 14.09636928[T] (600[M
X_acq_duration      = 0.69206016[s]
X_domain            = 13C
X_freq              = 150.91343039[MHz]
X_offset            = 100[ppm]
X_points            = 32768
X_prescans          = 4
X_resolution        = 1.44496109[Hz]
X_sweep             = 47.34848485[kHz]
Irr_domain          = 1H
Irr_freq            = 600.1723046[MHz]
Irr_offset          = 5[ppm]
Clipped             = FALSE
Mod_return          = 1
Scans               = 322
Total_scans         = 322

X_90_width          = 14.7[us]
X_acq_time          = 0.69206016[s]
X_angle             = 30[deg]
X_atn               = 2[dB]
X_pulse             = 4.9[us]
Irr_atn_dec         = 22.6[dB]
Irr_atn_noe         = 22.6[dB]
Irr_noise           = WALTZ
Decoupling          = TRUE
Initial_wait        = 1[s]
Noe                 = TRUE
Noe_time            = 2[s]
Recvr_gain          = 60
Relaxation_delay    = 2[s]
Repetition_time     = 2.69206016[s]
Temp_get            = 22.2[dC]
```

S 4

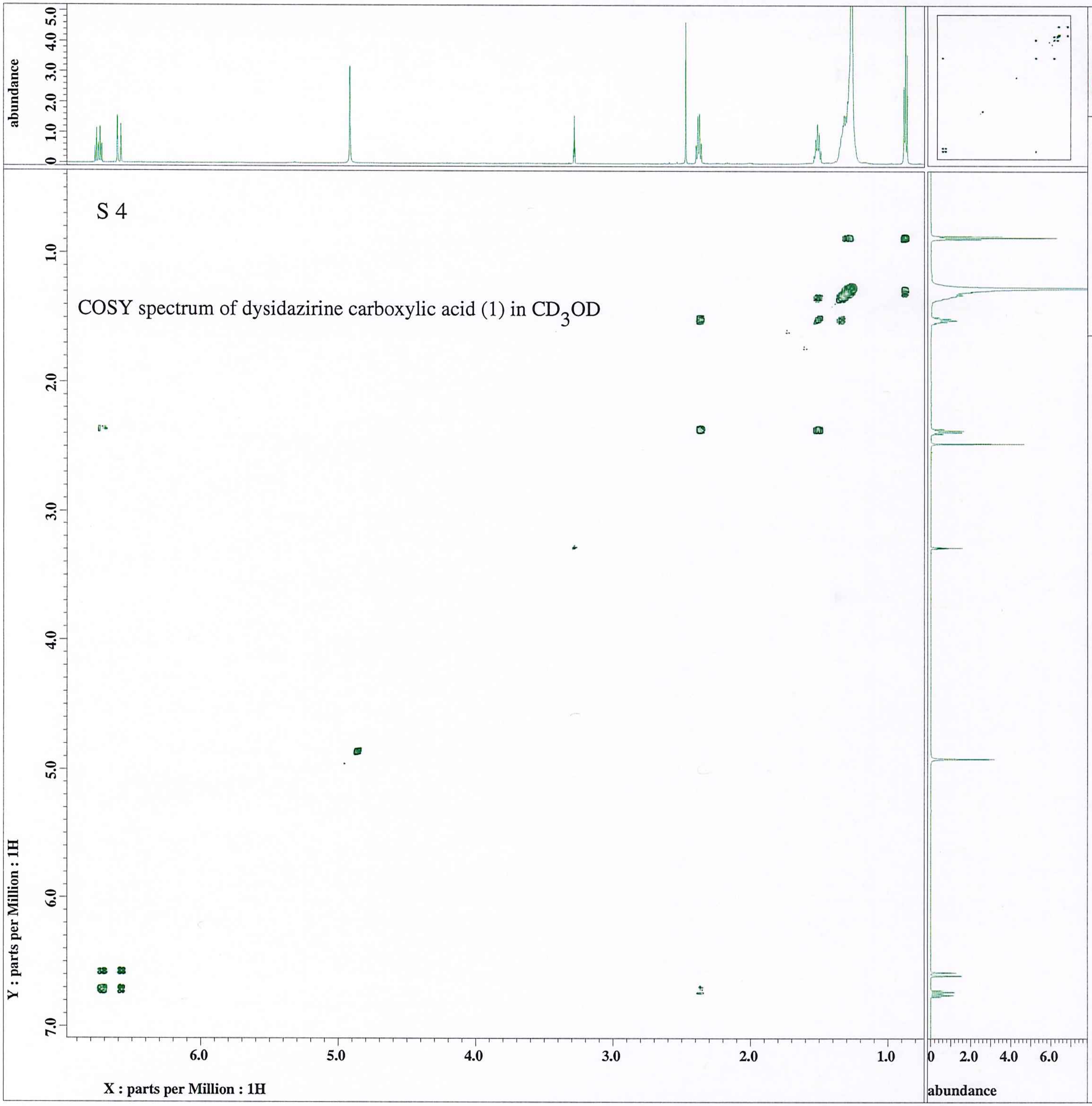

COSY spectrum of dysidazirine carboxylic acid (1) in CD_3OD

```
---- PROCESSING PARAMETERS ----
sinbell_auto
fft : 1 : TRUE : TRUE
ppm
[transpose]
zerofill : 4
sinbell_auto
fft : 1 : TRUE : TRUE
ppm
abs
thresh : 2[%] : 1
symmetrize : Cosy : 24
[transpose]

Filename          = 6sg73-9-dqf_cosy_pfg-
Author            = Smithsonian
Experiment        = dqf_cosy_pfg.ex2
Sample_id         = 6sg48-10
Solvent           = METHANOL-D3
Creation_time     = 27-MAR-2018 23:01:24
Revision_time     = 24-AUG-2021 15:05:41
Current_time      = 24-AUG-2021 15:06:38

Comment           = gradient absolute val
Data_format       = 2D REAL REAL
Dim_size          = 1024, 1024
Dim_title         = 1H 1H
Dim_units         = [ppm] [ppm]
Dimensions        = X Y
Site              = ECA 600
Spectrometer      = ECA600-AID

Field_strength    = 14.09636928[T] (600[M
X_acq_duration    = 0.2276352[s]
X_domain          = 1H
X_freq            = 600.1723046[MHz]
X_offset          = 3.66988[ppm]
X_points          = 1280
X_prescans        = 4
X_resolution      = 4.3929937[Hz]
X_sweep           = 5.62303194[kHz]
Y_domain          = 1H
Y_freq            = 600.1723046[MHz]
Y_offset          = 3.66988[ppm]
Y_points          = 256
Y_prescans        = 0
Y_resolution      = 17.56723332[Hz]
Y_sweep           = 4.49721173[kHz]
Irr_domain        = 1H
Irr_freq          = 600.1723046[MHz]
Irr_offset        = 5[ppm]
Tri_domain        = 1H
Tri_freq          = 600.1723046[MHz]
Tri_offset        = 5[ppm]
Clipped           = FALSE
Mod_return        = 1
Scans             = 4
Total_scans       = 1024

X_acq_time        = 0.2276352[s]
X_atn             = 3[dB]
X_pulse           = 7.9[us]
Y_acq_time        = 56.92416[ms]
Irr_mode          = Off
Tri_mode          = Off
Dante_presat      = FALSE
Grad_1            = 1[ms]
Grad_1_amp        = 60[mT/m]
```

S 5

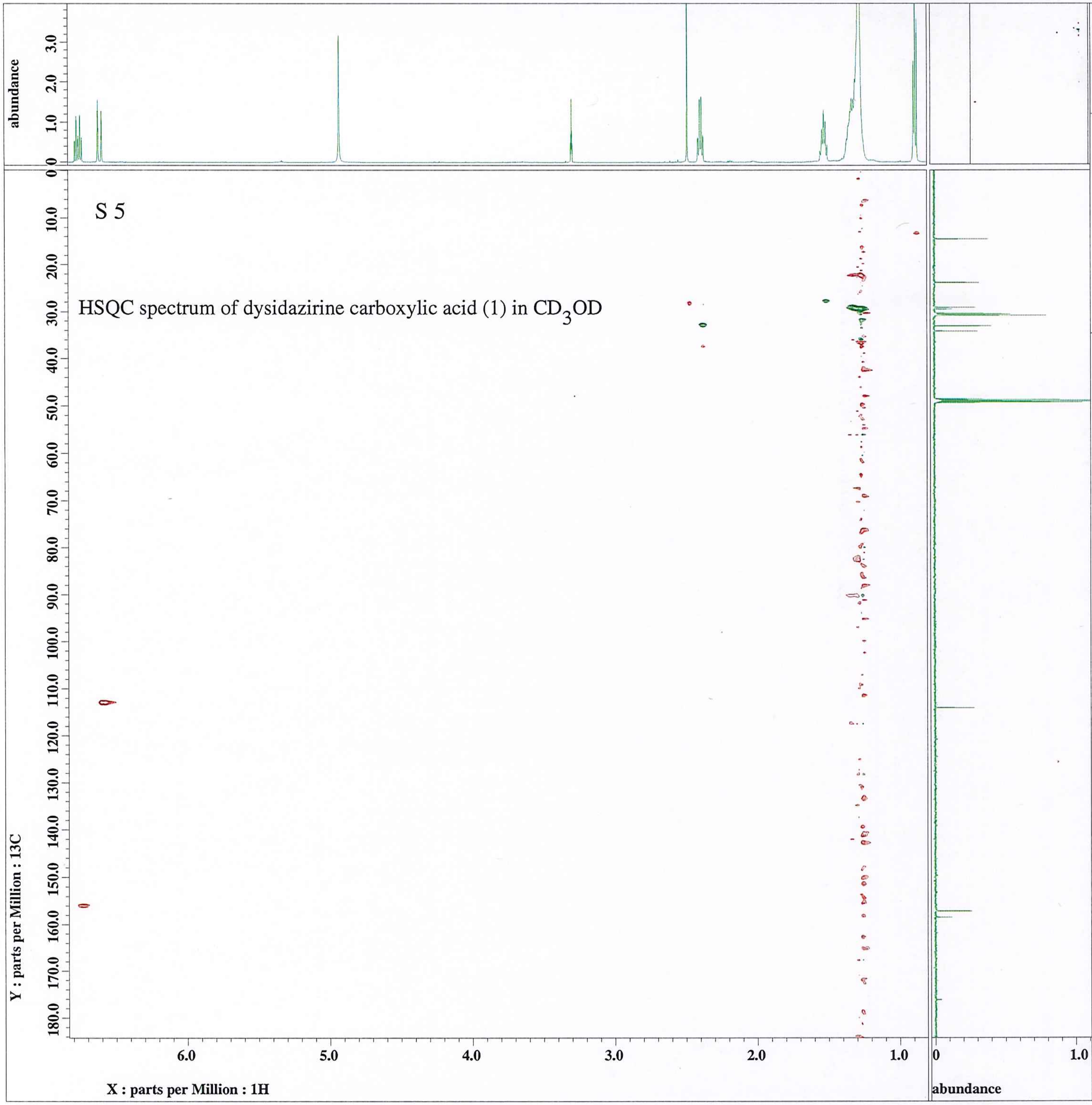

HSQC spectrum of dysidazirine carboxylic acid (1) in CD_3OD

```
---- PROCESSING PARAMETERS ----
dc_balance : 0 : FALSE
sexp : 2.0[Hz] : 0.0[s]
trapezoid3 : 0[%] : 80[%] : 100[%]
zerofill : 1
fft : 1 : TRUE : TRUE
machinephase
ppm

Filename            = 6sg73-9-hsqc_edit_dec
Author              = Smithsonian
Experiment          = hsqc_edit_dec_ph
Sample_id           = 6sg73-9
Solvent             = METHANOL-D3
Creation_time       = 19-APR-2018 11:35:24
Revision_time       = 24-AUG-2021 15:31:35
Current_time        = 24-AUG-2021 15:38:58

Comment             = absorption_hsqc
Data_format         = 2D COMPLEX COMPLEX
Dim_size            = 819, 512
Dim_title           = 1H 13C
Dim_units           = [ppm] [ppm]
Dimensions          = X Y
Site                = ECA 600
Spectrometer        = ECA600-AID

Field_strength      = 14.09636928[T] (600[M
X_acq_duration      = 0.165888[s]
X_domain            = 1H
X_freq              = 600.1723046[MHz]
X_offset            = 4.78513[ppm]
X_points            = 1024
X_prescans          = 4
X_resolution        = 6.02816358[Hz]
X_sweep             = 6.17283951[kHz]
Y_domain            = 13C
Y_freq              = 150.91343039[MHz]
Y_offset            = 91.91071[ppm]
Y_points            = 256
Y_prescans          = 0
Y_resolution        = 108.86984392[Hz]
Y_sweep             = 27.87068004[kHz]
Tri_domain          = 1H
Tri_freq            = 600.1723046[MHz]
Tri_offset          = 5[ppm]
Clipped             = FALSE
Mod_return          = 1
Scans               = 16
Total_scans         = 4096

X_acq_time          = 0.165888[s]
X_atn               = 3[dB]
X_pulse             = 7.9[us]
Y_acq_time          = 9.18528[ms]
Y_atn               = 2[dB]
Y_p1_correction     = 360.0
Y_pulse             = 14.7[us]
Irr_atn_dec         = 19.1[dB]
Irr_noise           = MPF8
Tri_mode            = Off
Composite_pulse     = FALSE
Dante_presat        = FALSE
```

S 6

HMBC spectrum of dysidazirine carboxylic acid (1) in CD_3OD

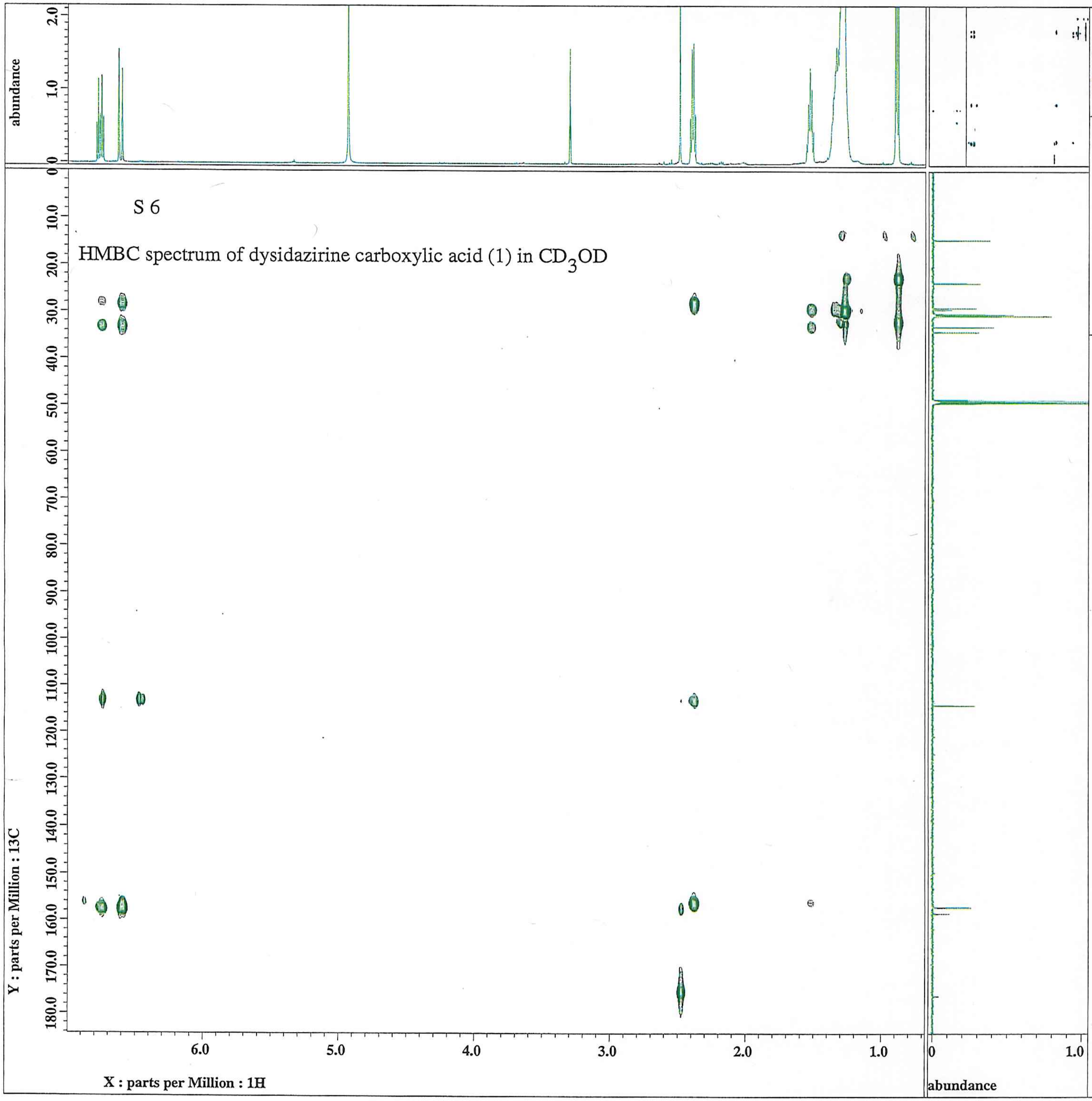


```
---- PROCESSING PARAMETERS ----
gauss : 5.0[Hz] : 0.0[s]
sinbell_auto
zerofill : 1
fft : 1 : TRUE : TRUE
ppm
[transpose]
sinbell4 : -60 : 160
trapezoid3 : 5[%] : 80[%] : 100[%]
zerofill : 2
fft : 1 : TRUE : TRUE
ppm
abs
[transpose]

Filename          = 6sg73-9-hmbc_pfg-3.jd
Author            = Smithsonian
Experiment        = hmbc_pfg.ex2
Sample_id         = 6sg73-9
Solvent           = METHANOL-D3
Creation_time     = 19-APR-2018 15:57:11
Revision_time     = 20-DEC-2021 10:25:17
Current_time      = 20-DEC-2021 10:26:24

Comment           = gradient enhanced HMB
Data_format       = 2D REAL REAL
Dim_size          = 1638, 512
Dim_title         = 1H 13C
Dim_units         = [ppm] [ppm]
Dimensions        = X Y
Site              = ECA 600
Spectrometer      = ECA600-AID

Field_strength    = 14.09636928[T] (600[M
X_acq_duration    = 0.331776[s]
X_domain          = 1H
X_freq            = 600.1723046[MHz]
X_offset          = 4.78513[ppm]
X_points          = 2048
X_prescans        = 4
X_resolution      = 3.01408179[Hz]
X_sweep           = 6.17283951[kHz]
Y_domain          = 13C
Y_freq            = 150.91343039[MHz]
Y_offset          = 91.91071[ppm]
Y_points          = 256
Y_prescans        = 0
Y_resolution      = 108.86984392[Hz]
Y_sweep           = 27.87068004[kHz]
Tri_domain        = 1H
Tri_freq          = 600.1723046[MHz]
Tri_offset        = 5[ppm]
Clipped           = FALSE
Mod_return        = 1
Scans             = 32
Total_scans       = 8192

X_acq_time        = 0.331776[s]
X_atn             = 3[dB]
X_pulse           = 7.9[us]
Y_acq_time        = 9.18528[ms]
Y_atn             = 2[dB]
Y_pulse           = 14.7[us]
Tri_mode          = Off
Dante_presat      = FALSE
Delta             = 62.5[ms]
Grad_1            = 1[ms]
Grad_1_amp        = 0.18[T/m]
Grad_2            = 1[ms]
```

S 7

Proton spectrum of dysidazirine (2) in $CDCl_3$ and CD_3OD

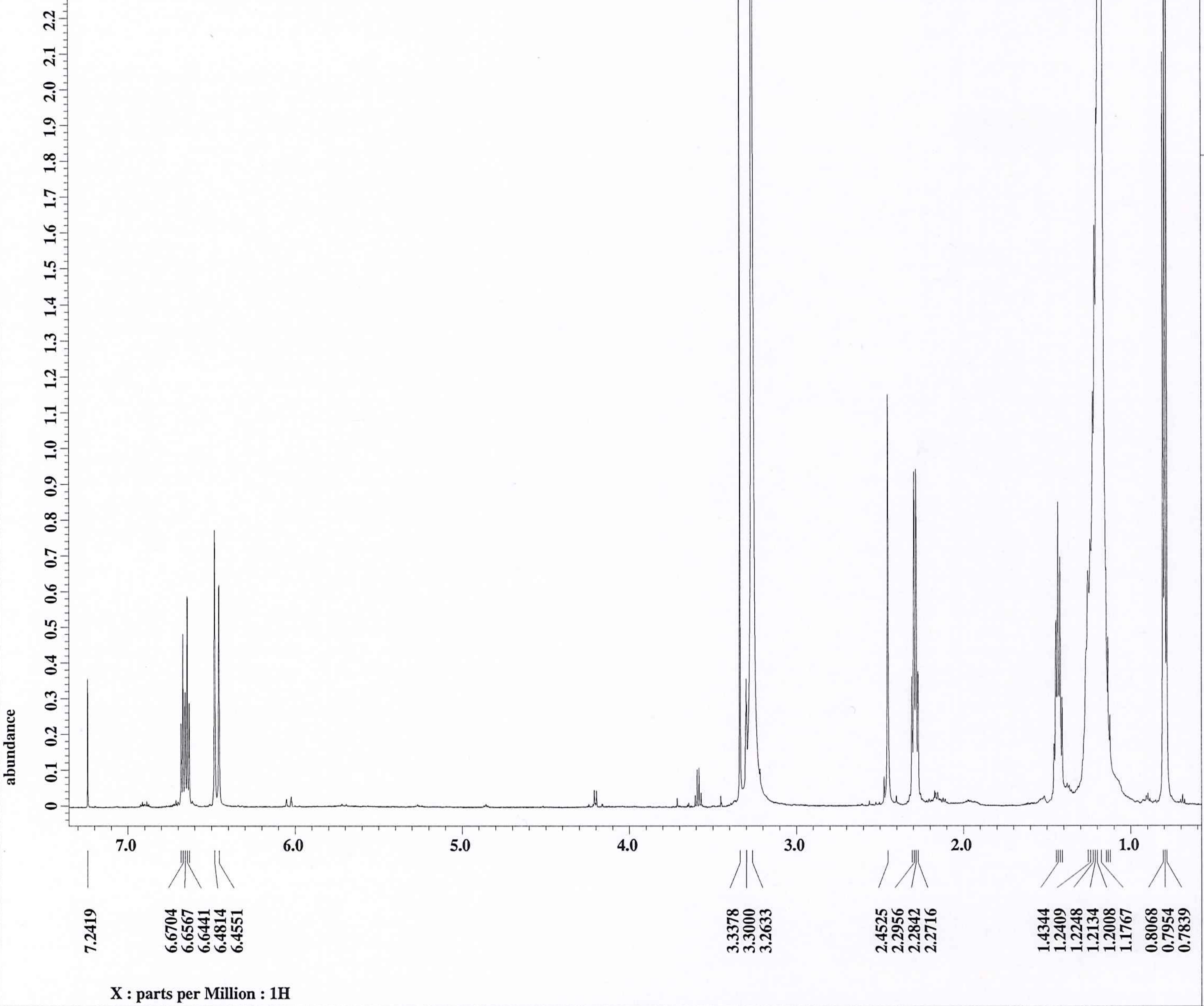


```
---- PROCESSING PARAMETERS ----
dc_balance : 0 : FALSE
sexp : 0.2[Hz] : 0.0[s]
trapezoid3 : 0[%] : 80[%] : 100[%]
zerofill : 1
fft : 1 : TRUE : TRUE
machinephase
ppm

Filename          = 6sg73-11-cdcle-cd3od-
Author            = Smithsonian
Experiment        = single_pulse.ex2
Sample_id         = 6sg73-11-cdcle-cd3od
Solvent           = CHLOROFORM-D
Creation_time     = 23-APR-2018 07:49:23
Revision_time     = 27-AUG-2021 11:01:47
Current_time      = 27-AUG-2021 11:02:05

Comment           = single_pulse
Data_format       = 1D COMPLEX
Dim_size          = 13107
Dim_title         = 1H
Dim_units         = [ppm]
Dimensions        = X
Site              = ECA 600
Spectrometer      = ECA600-AID

Field_strength    = 14.09636928[T] (600[M
X_acq_duration    = 1.4548992[s]
X_domain          = 1H
X_freq            = 600.1723046[MHz]
X_offset          = 5[ppm]
X_points          = 16384
X_prescans        = 1
X_resolution      = 0.68733284[Hz]
X_sweep           = 11.26126126[kHz]
Irr_domain        = 1H
Irr_freq          = 600.1723046[MHz]
Irr_offset        = 5[ppm]
Tri_domain        = 1H
Tri_freq          = 600.1723046[MHz]
Tri_offset        = 5[ppm]
Clipped           = FALSE
Mod_return        = 1
Scans             = 8
Total_scans       = 8

X_90_width        = 7.9[us]
X_acq_time        = 1.4548992[s]
X_angle           = 45[deg]
X_atn             = 3[dB]
X_pulse           = 3.95[us]
Irr_mode          = Off
Tri_mode          = Off
Dante_presat      = FALSE
Initial_wait      = 1[s]
Recvr_gain        = 42
Relaxation_delay  = 5[s]
Repetition_time   = 6.4548992[s]
Temp_get          = 22[dC]
```

S 8

Carbon spectrum of dysidazirine (2) in $CDCl_3$

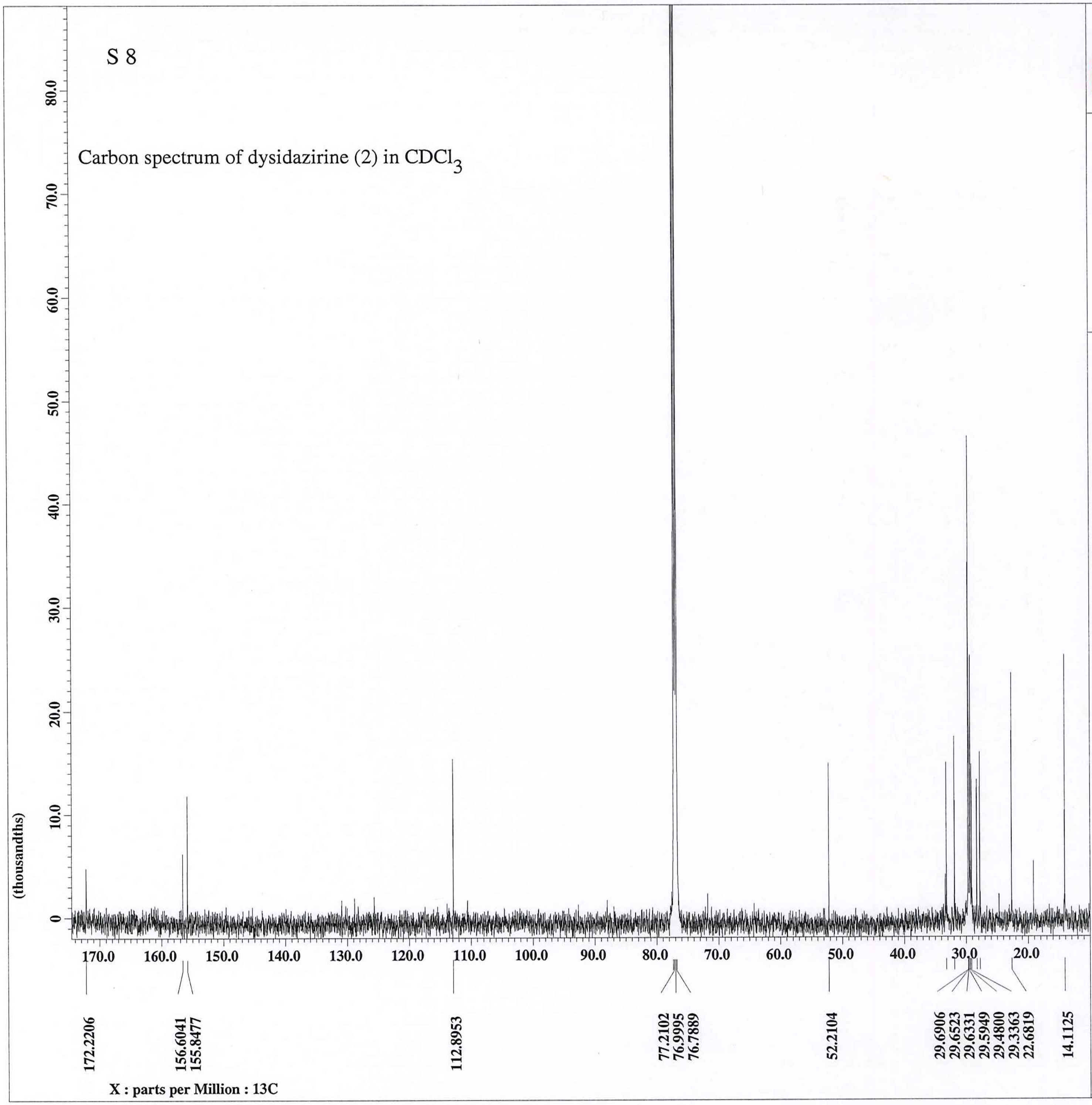


```
---- PROCESSING PARAMETERS ----
dc_balance : 0 : FALSE
sexp : 2.0[Hz] : 0.0[s]
trapezoid3 : 0[%] : 80[%] : 100[%]
zerofill : 1
fft : 1 : TRUE : TRUE
machinephase
ppm

Filename           = 6sg73-11-Me-cdcl3-sin
Author             = Smithsonian
Experiment         = single_pulse_dec
Sample_id          = 6sg73-11-Me-cdcl3
Solvent            = CHLOROFORM-D
Creation_time      = 27-APR-2018 00:07:48
Revision_time      = 27-AUG-2021 11:13:32
Current_time       = 27-AUG-2021 11:13:45

Comment            = single pulse decouple
Data_format        = 1D COMPLEX
Dim_size           = 26214
Dim_title          = 13C
Dim_units          = [ppm]
Dimensions         = X
Site               = ECA 600
Spectrometer       = ECA600-AID

Field_strength     = 14.09636928[T] (600[M
X_acq_duration     = 0.69206016[s]
X_domain           = 13C
X_freq             = 150.91343039[MHz]
X_offset           = 100[ppm]
X_points           = 32768
X_prescans         = 4
X_resolution       = 1.44496109[Hz]
X_sweep            = 47.34848485[kHz]
Irr_domain         = 1H
Irr_freq           = 600.1723046[MHz]
Irr_offset         = 5[ppm]
Clipped            = FALSE
Mod_return         = 1
Scans              = 22000
Total_scans        = 22000

X_90_width         = 14.7[us]
X_acq_time         = 0.69206016[s]
X_angle            = 30[deg]
X_atn              = 2[dB]
X_pulse            = 4.9[us]
Irr_atn_dec        = 22.6[dB]
Irr_atn_noe        = 22.6[dB]
Irr_noise          = WALTZ
Decoupling         = TRUE
Initial_wait       = 1[s]
Noe                = TRUE
Noe_time           = 2[s]
Recvr_gain         = 60
Relaxation_delay   = 2[s]
Repetition_time    = 2.69206016[s]
Temp_get           = 22.4[dC]
```

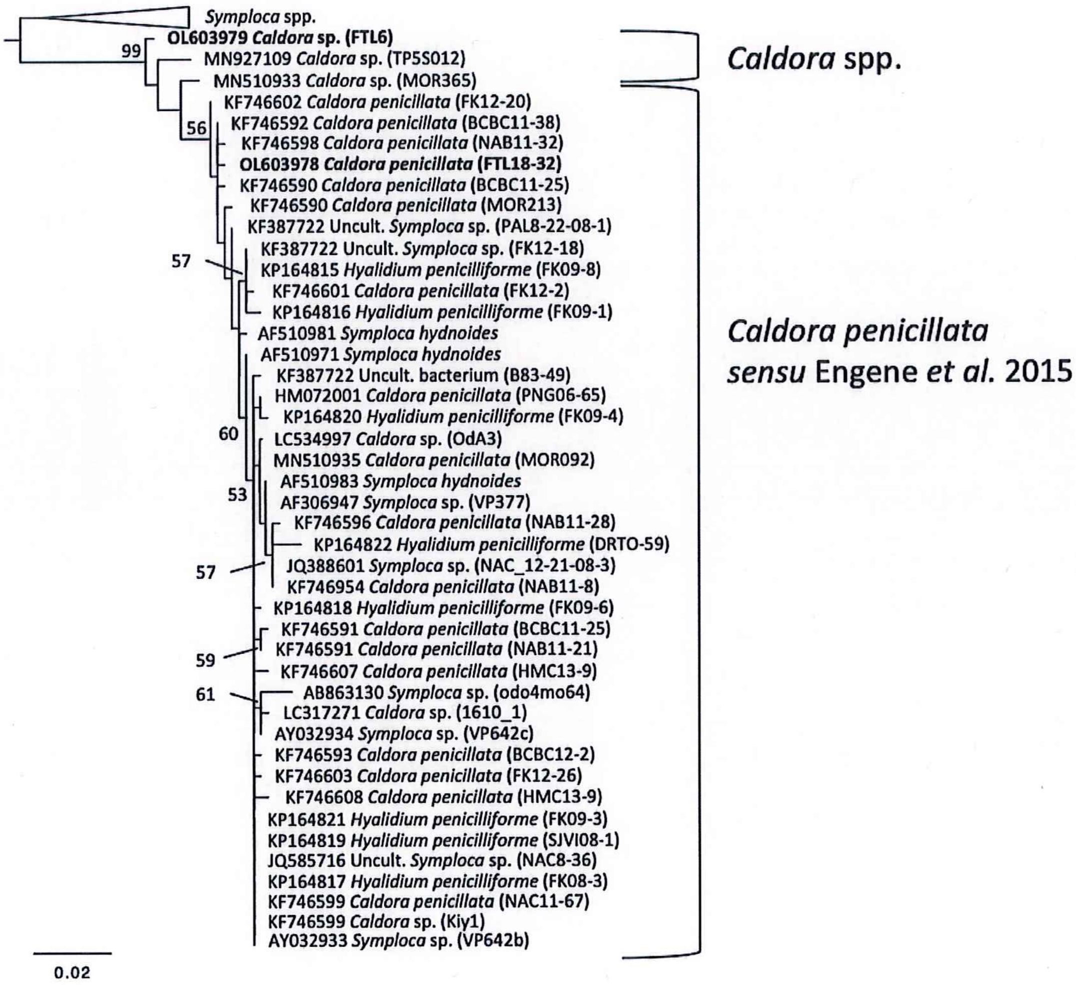

Supplemental Figure S9. Fully developed 16S rDNA maximum likelihood tree showing *Caldora penicillata* sequences summarized as a triangle in Figure 2. Note variation of sequences taxonomy found on Genbank corresponding to older names applied to this species prior to formal description of the genus and epithet combination by Engene et al. 2015. FTL18-32 was collected at the same Ft. Lauderdale location and represents the more widespread *Caldora penicillata*.
